# Molecular dynamics simulations of the interaction of wild type and mutant human CYP2J2 with polyunsaturated fatty acids

**DOI:** 10.1186/s13104-019-4797-8

**Published:** 2019-11-21

**Authors:** K. K. Abelak, D. Bishop-Bailey, I. Nobeli

**Affiliations:** 1 Comparative Biomedical Sciences, Royal Veterinary College, Royal College Street, London, NW1 0TU UK; 2 Department of Biological Sciences, Institute of Structural and Molecular Biology, Birkbeck, Malet Street, London, WC1E 7HX UK

**Keywords:** CYP2J2, Cytochrome P450, Polyunsaturated fatty acids, Molecular dynamics, Homology model, Docking, Arachidonic acid, Docosahexaenoic acid, Eicosapentaenoic acid, Polyunsaturated fatty acids

## Abstract

**Objectives:**

The data presented here is part of a study that was aimed at characterizing the molecular mechanisms of polyunsaturated fatty acid metabolism by CYP2J2, the main cytochrome P450 enzyme active in the human cardiovasculature. This part comprises the molecular dynamics simulations of the binding of three eicosanoid substrates to wild type and mutant forms of the enzyme. These simulations were carried out with the aim of dissecting the importance of individual residues in the active site and the roles they might play in dictating the binding and catalytic specificity exhibited by CYP2J2.

**Data description:**

The data comprise: (a) a new homology model of CYP2J2, (b) a number of predicted low-energy complexes of CYP2J2 with arachidonic acid, docosahexaenoic acid and eicosapentaenoic acid, produced with molecular docking and (c) a series of molecular dynamics simulations of the wild type and four mutants interacting with arachidonic acid as well as simulations of the wild type interacting with the two other eicosanoid ligands. The simulations may be helpful in identifying the determinants of substrate specificity of this enzyme and in unraveling the role of individual mutations on its function. They may also help guide the generation of mutants with altered substrate preferences.

## Objective

The polyunsaturated fatty acids (PUFAs) arachidonic acid (AA), docosahexaenoic acid (DHA) and eicosapentaenoic acid (EPA) are oxidised by cytochrome P450 (CYP) enzymes to produce metabolically active products that play significant roles in inflammation pathways [1, 2]. Due to the absence of a crystal structure of the main such enzyme in the human cardiovasculature (CYP2J2), the precise mechanism by which it metabolises PUFAs into specific stereo- and regio-epoxyisomers is not fully understood. Consequently, the effect of mutations in the protein sequence arising from non-synonymous single nucleotide polymorphisms found in the population cannot be predicted, hindering our ability to link genomic information to dysregulation of inflammatory responses and thus successful prognoses of cardiovascular health. In this project, we aimed to understand binding of PUFAs in the active site of CYP2J2 using computational methods and leverage this information to investigate the residues essential for ligand positioning and metabolism. In previous work, our groups investigated the interaction of AA with human CYP2J2 and revealed Arg117 as a key player in the recognition of this substrate [3], although these simulations were relatively short (50 ns). Simulations from other studies have come to diverse conclusions about the role of individual residues in the active site [4–6]. Here, we tried to investigate further using much more extensive simulations of both wild type and mutant forms of the enzyme. These new simulations confirmed the importance of Arg117 but in addition suggested Arg111 as a residue necessary for epoxidation and pointed to the role of two more arginine residues in the active site that allow some redundancy in substrate tethering and contribute to the flexibility of the catalytic capabilities of the system. Expression trials in HEK293T cells to produce CYP2J2 and its mutants were unsuccessful so the computationally derived hypotheses could not be validated in the lifetime of this project.

**Table 1:** Overview of data files/data sets

Label	Name of data file/data set	File types (file extension)	Data repository and identifier (DOI or accession number)
Data set 1: Homology modelling, molecular docking and molecular dynamics simulations of wild type and mutant human CYP2J2 with three polyunsaturated fatty acids	Abelak_etal_Methods.pdf C2J2_min3_mod_noH.pdb create_sim4_repeats.sh docking_wildtype_C2J2.zip	PDF document PDB file (.pdb) Shell script (.sh) Zipped file of 9 pdb files (.zip)	Zenodo 10.5281/zenodo.3465884
Data set 2: Molecular dynamics simulations of the interaction of wild type human CYP2J2 with arachidonic acid	MD_wt_CYP2J2_AA_StateX_repeatY.zip (X = pose number, 1 ≤ X ≤ 6; Y = repeat number, 1 ≤ Y ≤ 4)	Zipped files (.zip)	Zenodo Poses 1 and 2 10.5281/zenodo.3465590 Poses 3 and 4 10.5281/zenodo.3466692 Poses 5 and 6 10.5281/zenodo.3473886
Data set 3: Molecular dynamics simulations of the interaction of wild type human CYP2J2 with DHA (POSES 1–4)	MD_wt_CYP2J2_DHA_StateX_repeatY.zip (X = pose number, 1 ≤ X ≤ 4; Y = repeat number, 1 ≤ Y ≤ 3)	Zipped files (.zip)	Zenodo 10.5281/zenodo.3473909
Data set 4: Molecular dynamics simulations of the interaction of wild type human CYP2J2 with EPA (POSES 1–4)	MD_wt_CYP2J2_EPA_StateX_repeatY.zip (X = pose number, 1 ≤ X ≤ 4; Y = repeat number, 1 ≤ Y ≤ 3)	Zipped files (.zip)	Zenodo 10.5281/zenodo.3473927
Data set 5: Molecular dynamics simulations of the interaction of mutant human CYP2J2 (R111A) with arachidonic acid	MD_mutR111A_CYP2J2_AA_StateX_repeatY.zip (X = pose number, 1 ≤ X ≤ 6; Y = repeat number, 1 ≤ Y ≤ 3)	Zipped files (.zip)	Zenodo Poses 1–3 10.5281/zenodo.3483594 Poses 4-6: 10.5281/zenodo.3483966
Data set 6: Molecular dynamics simulations of the interaction of mutant human CYP2J2 (R117A) with arachidonic acid	MD_mutR117A_CYP2J2_AA_StateX_repeatY.zip (X = pose number, 1 ≤ X ≤ 6; Y = repeat number, 1 ≤ Y ≤ 3)	Zipped files (.zip),	Zenodo Poses 1–4 10.5281/zenodo.3482943 Poses 5-6: 10.5281/zenodo.3483493
Data set 7: Molecular dynamics simulations of the interaction of double mutant human CYP2J2 (R111A, R117A) with arachidonic acid	MD_mutR111A_R117A_CYP2J2_AA_StateX_repeatY.zip (X = pose number, 1 ≤ X ≤ 6; Y = repeat number, 1 ≤ Y ≤ 3)	Zipped files (.zip)	Zenodo Poses 1–3 10.5281/zenodo.3484029 Poses 4-6: 10.5281/zenodo.3484124
Data set 8: Molecular dynamics simulations of the interaction of quadruple mutant human CYP2J2 (R111A, R117A, R382A, R446A) with arachidonic acid	MD_quadmut_CYP2J2_AA_StateX_repeatY.zip (X = pose number, 1 ≤ X ≤ 6; Y = repeat number, 1 ≤ Y ≤ 3)	Zipped files (.zip)	Zenodo Poses 1–3 10.5281/zenodo.3484437 Poses 4-6: 10.5281/zenodo.3484448

## Data description

The data presented here comprise the results of homology modeling of the human wild type CYP2J2 and generation of models for a series of mutants [7]; molecular docking of three eicosanoid ligands (AA, DHA and EPA) to wild type CYP2J2 [7]; finally, a series of molecular dynamics simulations of the wild type and mutant enzyme with the three ligands [8–20]. Below is a brief description of each part of the data. More details are available in the Methods document on the top Zenodo repository [7].

### Homology model of CYP2J2

The homology model [7] is based on the UniProt [21] protein sequence with UID P51589. A model of the sequence with the N-terminal transmembrane domain (residues 1–43) trimmed was built using MODELLER version 9.14 [22], using as templates the PDB structures: 1SUO [23], 2P85 [24], 3EBS [25] and 1Z10 [26]. A haem molecule was incorporated into the model building using the HETATM records from PDB structure 1SUO.

Structure models of mutants of CYP2J2 were produced using the homology model of the wild type enzyme as the starting point and changing residues 111, 117, 382 and 446 from arginine to alanine. The expectation was that mutating these residues to a non-charged amino acid would have a noticeable impact on the binding of fatty acid substrates.

### Docking of PUFAs to CYP2J2

The fatty acids arachidonic acid (AA), docosahexaenoic acid (DHA) and eicosapentaenoic acid (EPA) were investigated in this study. The structure of AA was obtained from the Zinc Dock database version 12 [27]. Structures for DHA and EPA were derived using the Automated Topology Builder version 2.2 [28]. Docking of all ligands to CYP2J2 models was carried out using Autodock VINA version 1.1.2 [29]. Five independent docking runs were carried out for each ligand.

### Molecular dynamics simulations

MD simulations were carried out using AMBER14 [30] as described in the Methods document (data set 1 [7]). The simulations included the standard minimization, heating, equilibration and production phases. Six docked wild type CYP2J2-AA complexes were simulated in four independent runs, each lasting 1 μs [8–10]. Simulations of the mutant enzymes started from the same six docked poses of AA but each pose was simulated in three repeats, each lasting 500 ns. Two single mutants were investigated (Arg111Ala [13, 14], Arg117Ala [15, 16]) followed by a double mutant (Arg111Ala and Arg117Ala [17, 18]) and finally a quadruple mutant (Arg111Ala, Arg117Ala, Arg382Ala and Arg446Ala [19, 20]). Simulations of DHA [12] and EPA [11] were carried out starting from four docked poses, each simulation repeated three times and lasting 300 ns.

The simulations highlighted two residues in the active site (Arg111 and Arg117) that appear to play important roles in anchoring the carboxylate group of the substrate. Simulations also suggested that mutating any one of these two residues, results in enhancing the role of the other one as a hydrogen-bond donor, and that if both are mutated, two more arginine residues (Arg382 and Arg446) can partially make up for the missing charged groups in the active site.

## Limitations

As with all computational studies, the data here should be interpreted with care. The starting CYP2J2 structure used in these simulations is a homology model, i.e. a structure built in silico using information from related proteins whose structures have been deposited in the PDB. Although we have built the model using an alignment of multiple, carefully selected structures, it is possible that inaccuracies in the initial structure have affected the final simulations. Our molecular dynamics simulations (ranging from 900 ns to 4 μs) are, to the best of our knowledge, the longest carried out on human CYP2J2 and, in addition, multiple repeats using the same starting docked pose of the ligand were used to assess the robustness of observations to differences introduced by the random nature of the algorithm. Despite the length of these simulations and the evidence pointing to reasonable convergence in energy terms, simulations appeared to sample different conformations of the system, even when the same starting pose was used (in different repeats). These MD runs thus point towards a very flexible system that is better described as an ensemble of possible states, whose probability is affected by the substrate nature or mutations in the active site. Longer simulation times would have been useful in revealing whether convergence of the system to a few distinct conformations is possible, given enough simulation time. The haem molecule plays an important role in these simulations. Haem was modeled here in its penta-coordinated high-spin ferric form but the alternative highly reactive iron-oxygen species complex should be considered too. Finally, modeling a restricted part of this system around the haem molecule using a quantum mechanical (QM) model would be advisable. A joint QM/MM system could be setup that would offer a more realistic representation of how the intermediate complex between haem and substrate is formed.

## Data Availability

The data described in this Data Note can be freely and openly accessed on Zenodo. Please see Table 1 and reference list for details. The list of doi links is given below: Data set 1: 10.5281/zenodo.3465884 Data set 2:10.5281/zenodo.3465590; 10.5281/zenodo.3466692; 10.5281/zenodo.3473886 Data set 3: 10.5281/zenodo.3473909 Data set 4: 10.5281/zenodo.3473927 Data set 5: 10.5281/zenodo.3483594; 10.5281/zenodo.3483966 Data set 6: 10.5281/zenodo.3482943; 10.5281/zenodo.3483493 Data set 7: 10.5281/zenodo.3484029; 10.5281/zenodo.3484124 Data set 8: 10.5281/zenodo.3484437; 10.5281/zenodo.3484448.

## Abbreviations

AA: arachidonic acid
CYP: cytochrome P450
DHA: docosahexaenoic acid
EPA: eicosapentaenoic acid
MD: molecular dynamics
MM: molecular mechanics
PDB: Protein Data Bank
PUFA: polyunsaturated fatty acid
QM: quantum mechanical
